# Surveillance for variants of SARS-CoV-2 to inform risk assessments

**DOI:** 10.2471/BLT.23.290093

**Published:** 2023-09-28

**Authors:** Homa Attar Cohen, Samuel Mesfin, Juniorcaius Ikejezie, Zyleen Kassamali, Finlay Campbell, Sandra Adele, Noe Guinko, Friday Idoko, Bernadette Basuta Mirembe, Maria Elizabeth Mitri, Ingrid Nezu, Kazuki Shimizu, Ajong Brian Ngongheh, Nikola Sklenovska, Nicksy Gumede, Fausta Shakiwa Mosha, Basant Mohamed, Aura Corpuz, Richard Pebody, Marco Marklewitz, Lionel Gresh, Jairo A Mendez Rico, Kareena Hundal, Masaya Kato, Amarnath Babu, Brett N Archer, Olivier le Polain de Waroux, Maria D Van Kerkhove, Abdirahman Mahamud, Lorenzo Subissi, Boris I Pavlin

**Affiliations:** a World Health Organization (WHO) Health Emergencies Programme, WHO, Avenue Appia 20, 1211 Geneva, Switzerland.; b WHO Regional Office for Africa, Brazzaville, Congo.; c WHO Regional Office for the Eastern Mediterranean, Cairo, Egypt.; d WHO Regional Office for Europe, Copenhagen, Denmark.; e Pan American Health Organization, Washington D.C., United States of America.; f WHO Regional Office for the Western Pacific, Manila, Philippines.; g WHO Regional Office for South-East Asia, New Delhi, India.

## Abstract

Since the beginning of the coronavirus disease 2019 (COVID-19) pandemic, numerous severe acute respiratory syndrome coronavirus 2 (SARS-CoV-2) variants have emerged, some leading to large increases in infections, hospitalizations and deaths globally. The virus’s impact on public health depends on many factors, including the emergence of new viral variants and their global spread. Consequently, the early detection and surveillance of variants and characterization of their clinical effects are vital for assessing their health risk. The unprecedented capacity for viral genomic sequencing and data sharing built globally during the pandemic has enabled new variants to be rapidly detected and assessed. This article describes the main variants circulating globally between January 2020 and June 2023, the genetic features driving variant evolution, and the epidemiological impact of these variants across countries and regions. Second, we report how integrating genetic variant surveillance with epidemiological data and event-based surveillance, through a network of World Health Organization partners, supported risk assessment and helped provide guidance on pandemic responses. In addition, given the evolutionary characteristics of circulating variants and the immune status of populations, we propose future directions for the sustainable genomic surveillance of SARS-CoV-2 variants, both nationally and internationally: (i) optimizing variant surveillance by including environmental monitoring; (ii) coordinating laboratory assessment of variant evolution and phenotype; (iii) linking data on circulating variants with clinical data; and (iv) expanding genomic surveillance to additional pathogens. Experience during the COVID-19 pandemic has shown that genomic surveillance of pathogens can provide essential, timely and evidence-based information for public health decision-making.

## Résumé

Depuis le début de la pandémie de coronavirus survenue en 2019 (COVID-19), de nombreux variants du coronavirus 2 du syndrome respiratoire aigu sévère (SARS-CoV-2) sont apparus, certains entraînant une forte augmentation du nombre d'infections, d'hospitalisations et de décès dans le monde. L'impact du virus sur la santé publique dépend de nombreux facteurs, notamment l'émergence de nouveaux variants viraux et leur propagation à l'échelle mondiale. Par conséquent, la détection précoce et la surveillance des variants ainsi que la caractérisation de leurs effets cliniques sont essentielles pour évaluer leur risque pour la santé. La capacité sans précédent de séquençage du génome viral et de partage des données, capacité mise en place à l'échelle mondiale pendant la pandémie, a permis de détecter et d'évaluer rapidement de nouveaux variants. Le présent article décrit les principaux variants circulant dans le monde entre janvier 2020 et juin 2023, les caractéristiques génétiques à l'origine de leur évolution et leur impact épidémiologique dans les différents pays et régions. Ensuite, nous expliquerons comment l'intégration de la surveillance des variants génétiques aux données épidémiologiques et à la surveillance fondée sur les événements, par l'intermédiaire d'un réseau de partenaires de l'Organisation mondiale de la santé, a permis de faciliter l'évaluation des risques et de fournir des orientations sur les mesures à prendre en période de pandémie. En outre, compte tenu des caractéristiques évolutives des variants en circulation et de l'état immunitaire des populations, nous proposons des orientations futures pour une surveillance génomique durable des variants du SARS-CoV-2, au niveau tant national qu'international: (i) optimiser la surveillance des variants en incluant le suivi environnemental; (ii) coordonner l'évaluation en laboratoire de l'évolution des variants et du phénotype; (iii) établir un lien entre les données sur les variants en circulation et les données cliniques; et (iv) étendre la surveillance génomique à d'autres agents pathogènes. L'expérience de la pandémie de COVID-19 a mis en évidence que la surveillance génomique des agents pathogènes peut fournir en temps utile des informations essentielles fondées sur des preuves en vue de la prise de décisions en matière de santé publique.

## Resumen

Desde el inicio de la pandemia de la enfermedad por coronavirus de 2019 (COVID-19), han aparecido numerosas variantes del coronavirus de tipo 2 causante del síndrome respiratorio agudo severo (SRAS-CoV-2), algunas de las que han provocado un gran aumento de las infecciones, hospitalizaciones y muertes en todo el mundo. El impacto del virus en la salud pública depende de muchos factores, entre ellos la aparición de nuevas variantes víricas y su propagación mundial. En consecuencia, la detección y vigilancia tempranas de las variantes y la caracterización de sus efectos clínicos son vitales para evaluar su riesgo sanitario. La capacidad sin precedentes de secuenciación genómica viral y de intercambio de datos creada a nivel mundial durante la pandemia ha permitido detectar y evaluar rápidamente variantes nuevas. En este artículo se describen las principales variantes que circulan a nivel mundial entre enero de 2020 y junio de 2023, la característica genética que impulsa la evolución de las variantes y el impacto epidemiológico de estas variantes en los diferentes países y regiones. En segundo lugar, se informa de cómo la integración de la vigilancia de variantes genéticas con los datos epidemiológicos y la vigilancia basada en eventos, a través de una red de asociados de la Organización Mundial de la Salud, apoyó la evaluación de riesgos y ayudó a proporcionar orientación sobre las respuestas a la pandemia. Además, dadas las características evolutivas de las variantes circulantes y el estado inmunitario de las poblaciones, se proponen orientaciones futuras para la vigilancia genómica sostenible de las variantes del SRAS-CoV-2, tanto a nivel nacional como internacional: (i) optimizar la vigilancia de las variantes mediante la inclusión de la monitorización ambiental; (ii) coordinar la evaluación de laboratorio de la evolución y el fenotipo de las variantes; (iii) vincular los datos sobre las variantes circulantes con los datos clínicos; y (iv) ampliar la vigilancia genómica a patógenos adicionales. La experiencia durante la pandemia de la COVID-19 ha demostrado que la vigilancia genómica de patógenos puede proporcionar información esencial, oportuna y basada en evidencias para la toma de decisiones en materia de salud pública.

## ملخص

منذ بداية جائحة مرض فيروس كورونا 2019 (كوفيد 19)، ظهرت العديد من متغيرات المتلازمة التنفسية الحادة الوخيمة لفيروس كورونا 2 (SARS-CoV-2)، وأدى بعضها إلى زيادات كبيرة في حالات العدوى، وحالات دخول المستشفى للعلاج، والوفيات على مستوى العالم. ويعتمد تأثير الفيروس على الصحة العامة على العديد من العوامل، بما في ذلك ظهور متغيرات فيروسية جديدة، وانتشارها على مستوى العالم. وبالتالي، فإن الكشف المبكر عن المتغيرات، ورصدها، وتوصيف آثارها الإكلينيكية، يعد أمر حيوي لتقييم مخاطرها الصحية. وقد أدت القدرة غير المسبوقة للتسلسل الجينومي الفيروسي، وتبادل البيانات التي تم بناؤها عالميًا أثناء الجائحة، إلى تمكين اكتشاف المتغيرات الجديدة وتقييمها بسرعة. تصف هذه المقالة المتغيرات الرئيسية المنتشرة عالميًا بين يناير/كانون ثاني 2020 ويونيو/حزيران 2023، والسمة الجينية التي تحفز تطور المتغيرات والتأثير الوبائي لهذه المتغيرات عبر الدول والمناطق. ثانيا، نحن نوضح كيف أن دمج رصد المتغيرات الجينية، مع البيانات الوبائية والرصد على أساس الأحداث، من خلال شبكة من شركاء منظمة الصحة العالمية، استطعنا دعم تقييم المخاطر وساعدنا في تقديم التوجيهات حول الاستجابات للجائحة. بالإضافة إلى ذلك، وبالنظر إلى الخصائص التطورية للمتغيرات المنتشرة، وحالة مناعية السكان، فإننا نقترح اتجاهات مستقبلية للرصد الجينومي المستدام لمتغيرات فيروس SARS-CoV-2، على الصعيدين الوطني والدولي: (1) تحسين رصد المتغيرات من خلال تضمين المراقبة البيئية؛ و(2) تنسيق التقييم المختبري لتطور المتغير والنموذج الظاهري؛ و(3) ربط بيانات المتغيرات المنتشرة بالبيانات الإكلينيكية؛ و(4) توسيع الرصد الجينومي لتشمل مسببات الأمراض الإضافية. أظهرت التجربة خلال جائحة كوفيد 19 أن الرصد الجينومي لمسببات الأمراض يمكن أن يوفر معلومات أساسية، وفي حينها، وقائمة على الأدلة، لاتخاذ القرارات المتعلقة بالصحة العامة.

## 摘要

自新型冠状病毒 (COVID-19) 疫情开始以来，出现了许多严重的急性呼吸综合征冠状病毒 2 (SARS-CoV-2) 变异株，其中一些变异株导致全球感染、住院和死亡人数大幅增加。该病毒对公共卫生的影响取决于许多因素，包括新病毒变异株的出现及其在全球的传播。因此，早期检测和监测变异株及其临床效果对于评估其卫生风险至关重要。疫情期间，在全球范围内建立了前所未有的病毒基因组测序和数据共享能力，使得新的变异株能够得到快速检测和评估。本文介绍了 2020 年 1 月至 2023 年 6 月期间在全球范围内流行的主要变异株、驱动变异株进化的遗传特征以及这些变异株在不同国家和地区的流行病学影响。其次，我们报告了如何通过世界卫生组织合作伙伴网络，将遗传变异监测与流行病学数据和基于事件的监测相结合，以支持风险评估并帮助提供疫情应对指南。此外，鉴于流行变异株的进化特征和人群的免疫状态，我们提出了在国内和国际范围内对 SARS-CoV-2 变异株进行可持续基因组监测的未来方向：(i) 通过纳入环境监测来优化变异株监测；(ii) 综合变异株进化和表型的实验室评估；(iii) 将流行变异株的数据与临床数据联系起来；以及 (iv) 将基因组监测范围扩大到其它病原体。新冠肺炎疫情期间的经验表明，病原体的基因组监测可以为公共卫生决策提供必要、及时和基于事实证据的信息。

## Резюме

С начала пандемии коронавирусной инфекции 2019 года (COVID-19) появилось множество вариантов тяжелого острого респираторного синдрома, вызываемого коронавирусом 2 (SARS-CoV-2), и некоторые из них привели к значительному росту числа заражений, случаев госпитализации и смертности во всем мире. Влияние вируса на общественное здоровье зависит от многих факторов, в том числе от появления новых вариантов вируса и их распространения по всему миру. Следовательно, раннее выявление этих вариантов и наблюдение за ними, а также характеристика их клинических эффектов являются жизненно важными при оценке риска для здоровья. Благодаря уникальным возможностям секвенирования вирусных геномов и обмена данными, созданным во время пандемии в глобальном масштабе, удалось быстро обнаружить и оценить новые варианты вируса. В данной статье описаны основные варианты, распространенные в мире в период с января 2020 года по июнь 2023 года, генетическая особенность, определяющая эволюцию форм, и эпидемиологическое воздействие этих форм в разных странах и регионах. Во-вторых, сообщается, как интеграция эпиднадзора за генетическими вариантами с эпидемиологическими данными и эпиднадзором на основе событий с помощью сети партнеров Всемирной организации здравоохранения способствовала оценке рисков и помогла разработать рекомендации по ответным мерам на пандемию. Кроме того, с учетом эволюционных характеристик циркулирующих вариантов и иммунного статуса населения предложены перспективные направления устойчивого геномного надзора за вариантами SARS-CoV-2 на национальном и международном уровнях: (i) оптимизация надзора за вариантами путем включения мониторинга окружающей среды; (ii) координация лабораторной оценки эволюции и фенотипа вариантов; (iii) увязка данных о циркулирующих вариантах с клиническими данными; (iv) расширение геномного надзора на другие патогены. Опыт во время пандемии COVID-19 свидетельствует о том, что геномный надзор за патогенами может предоставить важную, своевременную и основанную на фактах информацию для общественного здравоохранения.

## Introduction

Genomic surveillance of pathogens is increasingly used to gain insights into emerging diseases.^1^ In particular, global genomic surveillance of severe acute respiratory syndrome coronavirus 2 (SARS-CoV-2) and its newly emerging variants has become essential for tackling uncertainties about the virus’s characteristics and its evolution. Despite disparities between countries, international efforts to build surveillance capacity have enabled viral sequences to be shared publicly at an unprecedented scale and timeliness, thereby facilitating the near-real-time monitoring of viral variants and their associated public health risks.^2^^–^^8^ However, since January 2022, many countries have changed their SARS-CoV-2 testing and sequencing strategies, which could compromise the effective detection, characterization and monitoring of current and emerging variants and their circulation patterns.

Here, we first describe insights gained from the availability of data on viral variants, including data from the genetic assessment of emerging variants and data on how different variants have spread geographically and co-circulated with, or replaced, other variants. The ability of a new variant to replace existing variants or even a whole generation of co-circulating variants is analysed in light of evolutionary genetic patterns, such as the identification of specific amino acid substitutions that drive, at least in part, increased viral transmissibility through convergent evolution (i.e. the process by which the same nucleotide or amino acid change occurs in multiple variants, despite these variants not being direct descendants of each other). In addition, we report insights into the evolutionary trajectory of SARS-CoV-2 in response to changing selection pressures, such as greater population immunity due to natural infection, vaccination or their combination (i.e. hybrid immunity).^9^ Second, we describe an integrated approach to health risk assessment that encompasses the early detection and characterization of new variants and their international impact. Finally, we suggest ways of ensuring the availability and sustainability of global variant surveillance in the future, and propose four specific areas in which efforts to maintain and improve genomic surveillance capacity should be focused nationally, regionally and globally.

## Genomic sequence sharing

The first complete SARS-CoV-2 genome sequence was shared with the World Health Organization (WHO) on 11 January 2020, and publicly submitted to Genbank on 12 January 2020.^10^^,^^11^ Up to 30 June 2023, over 15.8 million SARS-CoV-2 sequences from 216 territories and countries have been shared publicly through the Global Initiative on Sharing All Influenza Data (GISAID).^12^ This unprecedented effort to sequence and share genomic surveillance data in near-real-time has revolutionized the way we acquire and interpret genomic data and incorporate it into disease risk assessment. For comparison, genomic sequence data on the influenza virus was first shared through GISAID in 1970. However, genomic surveillance of the influenza virus has a different objective and sampling strategy to SARS-CoV-2. The number of shared viral sequences from human samples was 589 in 1970, 8376 in 2010 and 14 502 in 2020.^12^

Countries may have different approaches to disseminating genomic sequence information. South Africa, for instance, publicly shared information on the transmission and immune escape properties of newly emerging variants (later designated Beta and Omicron) and on their associated disease severity a few days after they were detected. This information helped WHO and its technical advisory group on SARS-CoV-2 virus evolution, as well as other countries, to prepare for the wave of infection caused by these variants of concern, and to adapt recommended public health measures accordingly. However, early reporting by South Africa had negative repercussions for the country’s economy because some other countries imposed travel restrictions.

## Surveillance and analysis

**Fig. 1:**
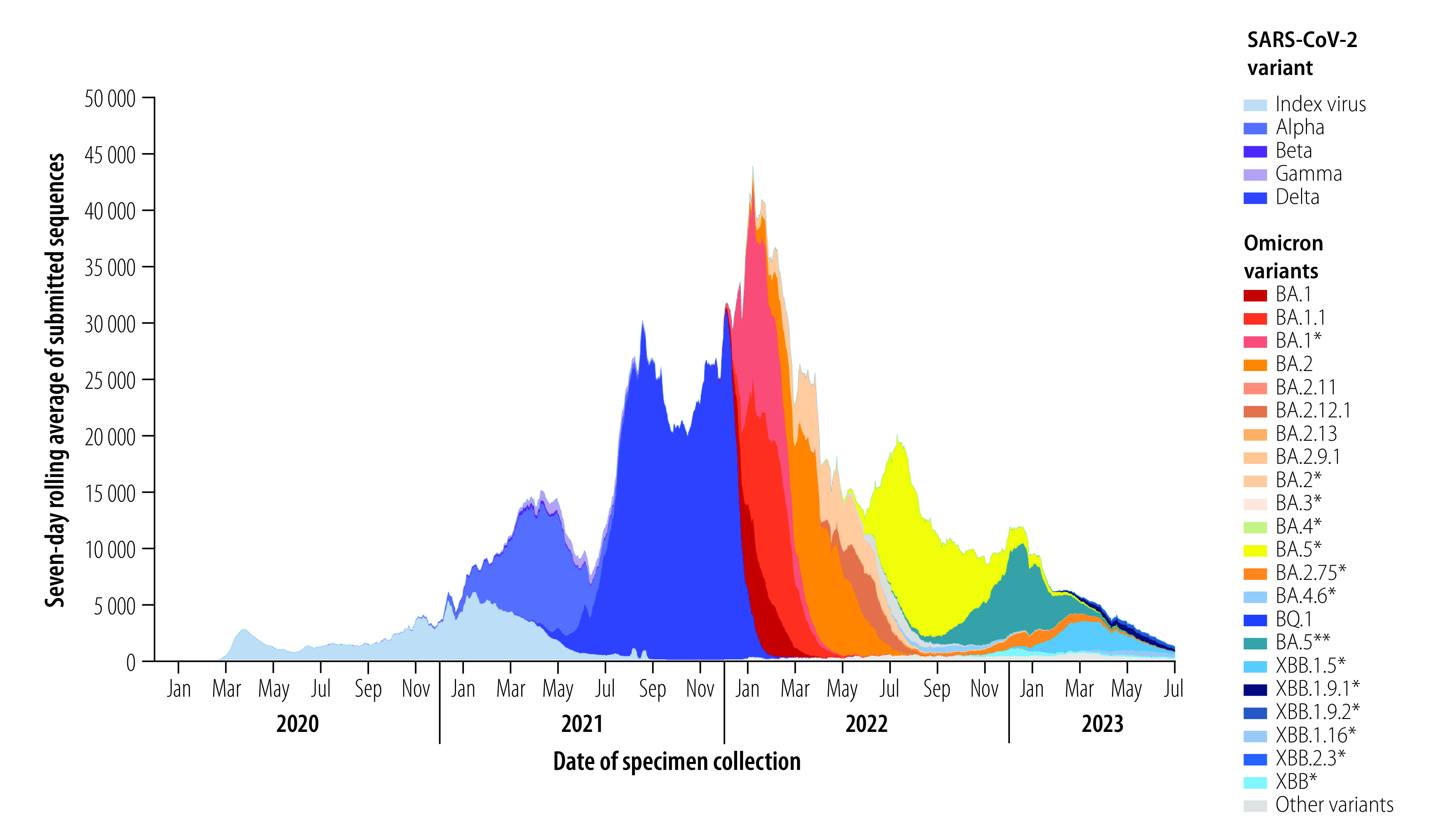
Emergence and scale of circulating SARS-CoV-2 variants of concern reported to the Global Initiative on Sharing All Influenza Data,^12^ globally, 1 January 2020 to 30 June 2023

**Fig. 2:**
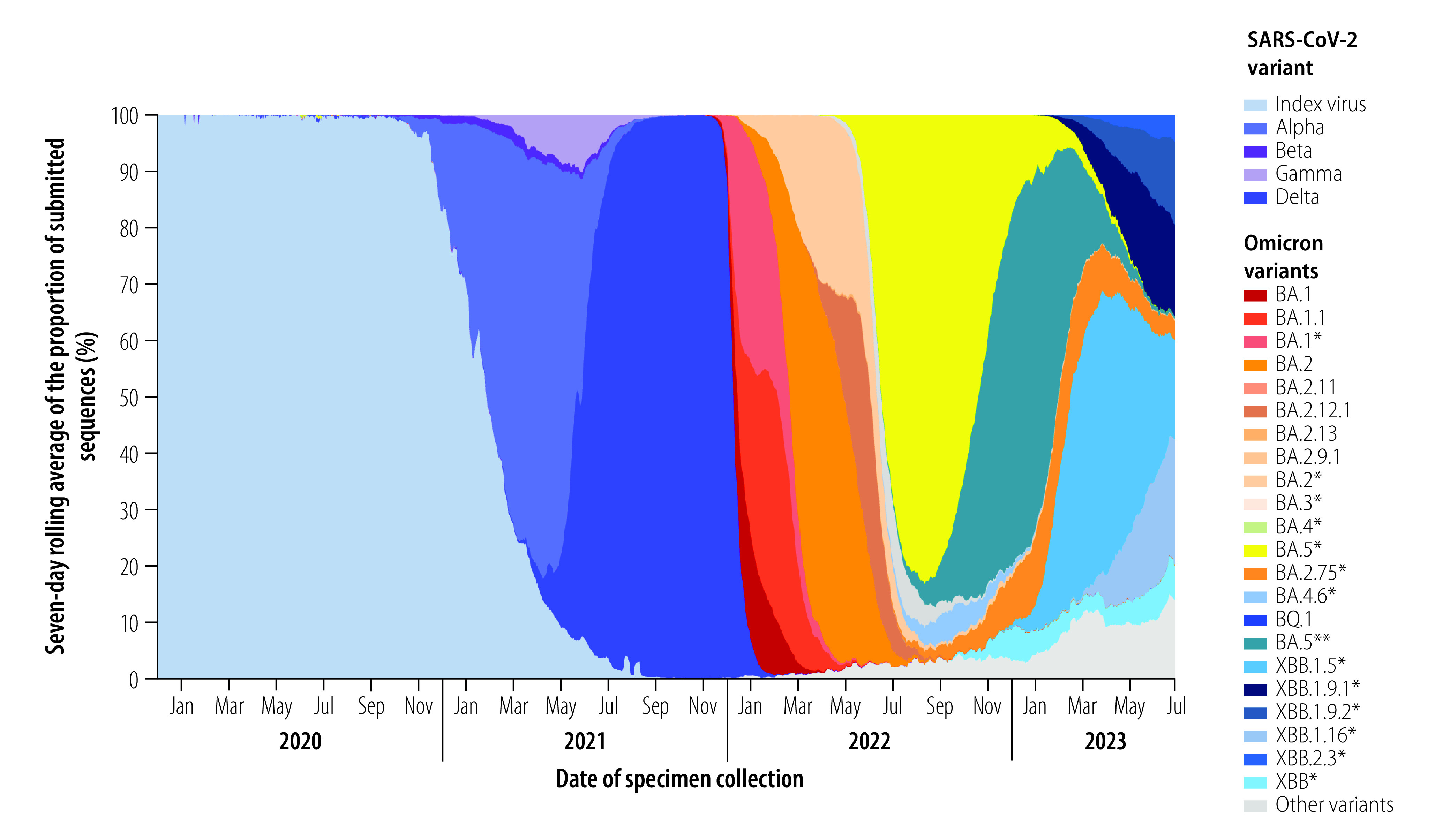
Emergence and replacement patterns of circulating SARS-CoV-2 variants of concern reported to the Global Initiative on Sharing All Influenza Data,^12^ by proportion of all circulating variants, globally, 1 January 2020 to 30 June 2023

The sequential emergence of viral variants and the global spread of SARS-CoV-2 between January 2020 and June 2023, as indicated by the number of viral genomic sequences submitted to GISAID, are illustrated in Fig. 1. The graph, which was based on genomic surveillance data shared by various countries, shows when different variants of concern and Omicron-descendent variants emerged and replaced previous variants. The criteria for assessing and naming variants of concern have been outlined previously, and a multitude of studies have reported on the molecular and biological characterization of specific variant lineages.^13^^,^^14^ In terms of population genetics, the Alpha, Beta and Gamma variants emerged as independent lineages (i.e. they were phylogenetically distinct from the index virus and from one another): they each exhibited different patterns of local, regional and global spread, of transmissibility and of co-circulation with other variants, and each had a different capacity to replace prevailing variants. The Alpha, Beta and Gamma variants co-circulated globally before the emergence of the Delta variant, which replaced all previously circulating variants and became dominant in the six WHO regions by July 2021 (Fig. 1). Similarly, the first Omicron variant rapidly replaced Delta worldwide by January 2022. The increase in the number of different Omicron genomic sequences reported after January 2022 (Fig. 1 and Fig. 2) reflects both the increasing number of cases of infection with each successive variant of concern (i.e. each successive variant had higher intrinsic or extrinsic transmissibility and a greater fitness advantage), and the global expansion in sequencing capacity and data sharing.

SARS-CoV-2: severe acute respiratory syndrome coronavirus 2.

Notes: The figure illustrates the time of emergence of SARS-CoV-2 variants and 7-day rolling averages of the number of SARS-CoV-2 genomic sequences reported to the Global Initiative on Sharing All Influenza Data (GISAID) for all five variants of concern being monitored, including Omicron sister lineages BA.1 to BA.5, Omicron recombinant sublineage XBB, pooled descendent lineages and other Omicron subvariants. The terms BA.1*, BA.2*, BA.3*, BA.4*, BA.5* and XBB* include all BA.1, BA.2, BA.3, BA.4, BA.5 and XBB pooled descendent lineages, respectively, except for subvariants reported individually. The category ‘other variants’ includes all other lineages not already listed, and unassigned lineages with a pending Pango name assignment, which were mostly poor-quality sequences that were difficult to assign. Data are based on metadata from GISAID available as at 30 June 2023. Variants with low sequence coverage were excluded.

SARS-CoV-2: severe acute respiratory syndrome coronavirus 2.

Notes: The figure illustrates the time of emergence of SARS-CoV-2 variants and 7-day rolling averages of the proportion of SARS-CoV-2 genomic sequences reported to the Global Initiative on Sharing All Influenza Data (GISAID) for all five variants of concern being monitored, including the Omicron sister lineages BA.1 to BA.5, Omicron recombinant sublineage XBB, pooled descendent lineages and other Omicron subvariants. The terms BA.1*, BA.2*, BA.3*, BA.4*, BA.5* and XBB* include all BA.1, BA.2, BA.3, BA.4, BA.5 and XBB pooled descendent lineages, respectively, except for subvariants reported individually. The category ‘other variants’ includes all other lineages not already listed and unassigned lineages with a pending Pango name assignment, which were mostly poor-quality sequences that were difficult to assign. Data are based on GISAID metadata available as at 30 June 2023. Variants with low sequence coverage were excluded.

**Fig. 3:**
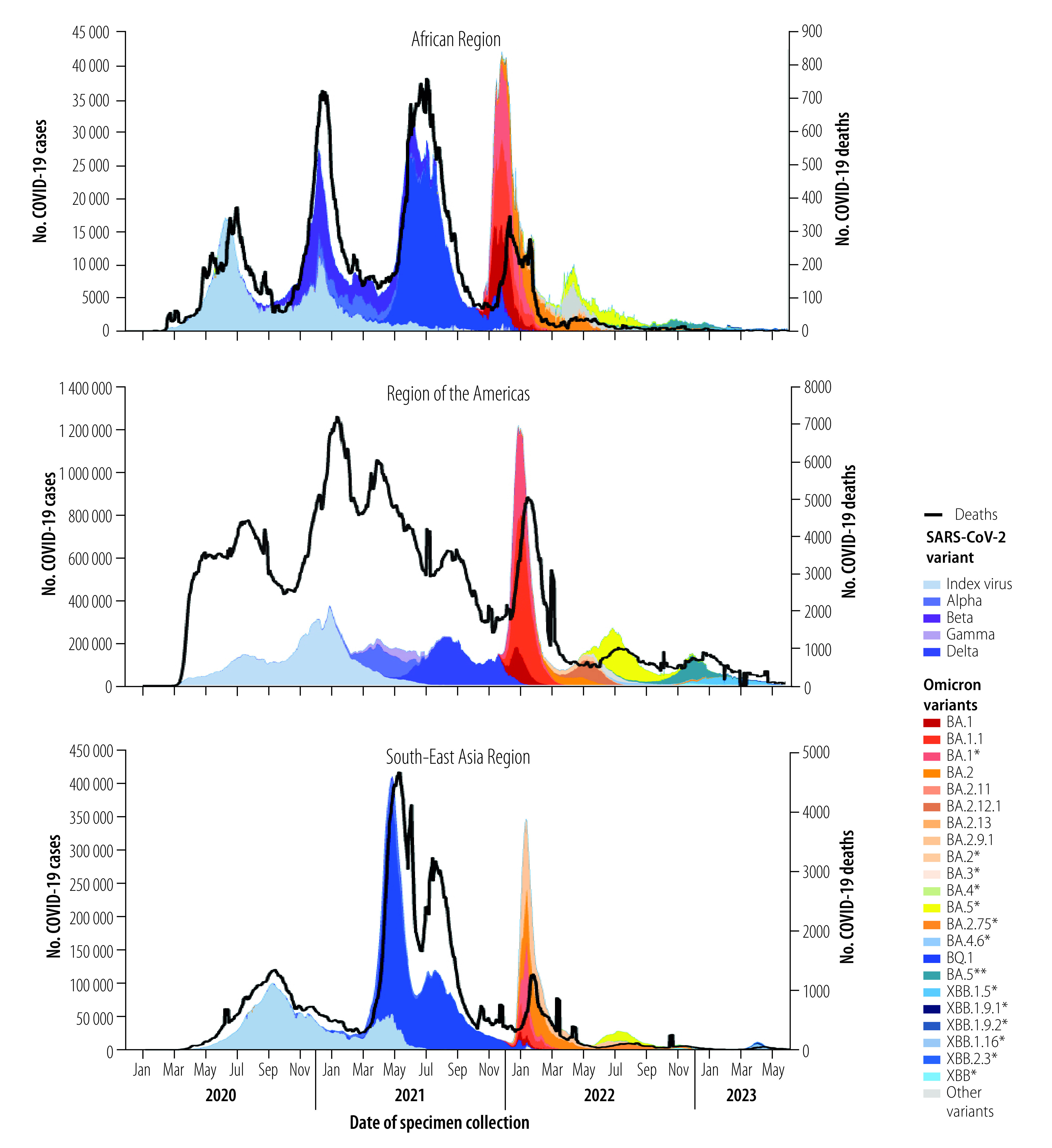
COVID-19 cases and deaths, African, Americas and South-East Asia Regions, 1 January 2020 to 30 June 2023

COVID-19: coronavirus disease 2019; SARS-CoV-2: severe acute respiratory syndrome coronavirus 2; WHO: World Health Organization.

Notes: The figure shows 7-day rolling averages of the number of new cases and deaths due to coronavirus disease 2019 (COVID-19). Data on cases and deaths were obtained from daily reports by WHO Member States, which were compiled, curated and validated daily by WHO. Severe acute respiratory syndrome coronavirus-2 sequence data were based on Global Initiative on Sharing All Influenza Data metadata available at 30 June 2023. Variants of concern being monitored included the Omicron sister lineages BA.1 to BA.5, Omicron recombinant sublineage XBB, pooled descendent lineages and other Omicron subvariants. The terms BA.1*, BA.2*, BA.3*, BA.4*, BA.5* and XBB* include all BA.1, BA.2, BA.3, BA.4, BA.5 and XBB pooled descendent lineages, respectively, except for subvariants reported individually. The category ‘other variants’ includes all other lineages not already listed and unassigned lineages with a pending Pango name assignment, which were mostly poor-quality sequences that were difficult to assign.

**Fig. 4:**
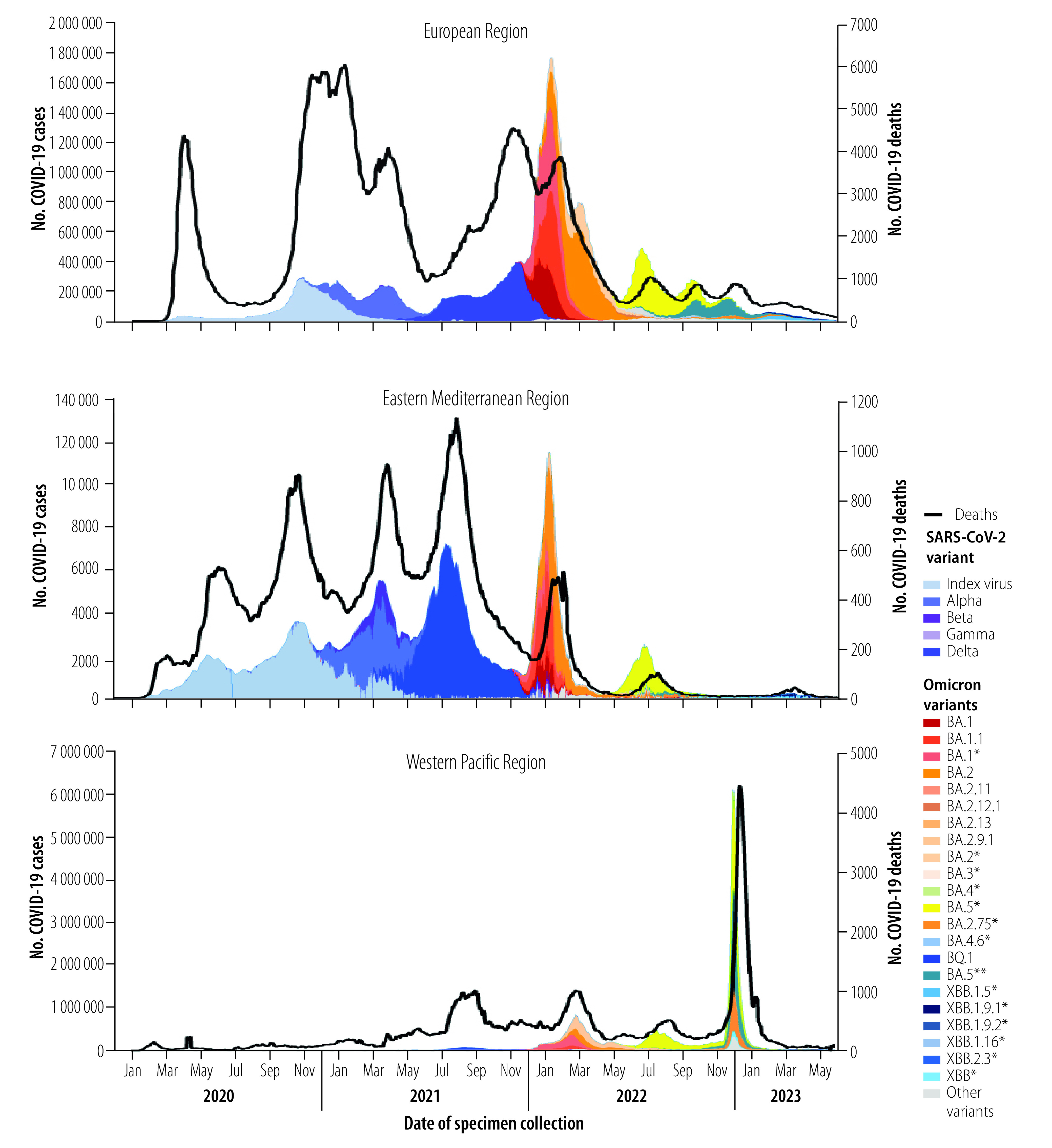
COVID-19 cases and deaths, European, Eastern Mediterranean and Western Pacific Regions, 1 January 2020 to 30 June 2023

COVID-19: coronavirus disease 2019; SARS-CoV-2: severe acute respiratory syndrome coronavirus 2; WHO: World Health Organization.

Notes: The figure shows 7-day rolling averages of the number of new cases and deaths due to coronavirus disease 2019 (COVID-19). Data on cases and deaths were obtained from daily reports by WHO Member States, which were compiled, curated and validated daily by WHO. Severe acute respiratory syndrome coronavirus-2 sequence data were based on Global Initiative on Sharing All Influenza Data metadata available at 30 June 2023. Variants of concern being monitored included the Omicron sister lineages BA.1 to BA.5, Omicron recombinant sublineage XBB, pooled descendent lineages and other Omicron subvariants. The terms BA.1*, BA.2*, BA.3*, BA.4*, BA.5* and XBB* include all BA.1, BA.2, BA.3, BA.4, BA.5 and XBB pooled descendent lineages, respectively, except for subvariants reported individually. The category ‘other variants’ includes all other lineages not already listed and unassigned lineages with a pending Pango name assignment, which were mostly poor-quality sequences that were difficult to assign.

In contrast to the genetic diversity exhibited by earlier variants of concern, most Omicron-descendent lineages differed by one or only a few mutations. Nevertheless, successive Omicron variants had the capacity to drive a new or renewed rise in the prevalence of the variant, thereby replacing former variants (Fig. 2) and causing a surge in cases, hospitalizations and deaths in many regions (Fig. 3 and Fig. 4). The cases illustrated in Fig. 3, and Fig. 4 are those reported officially to WHO by Member States. However, case definitions and identification strategies may have differed between countries or within a single country during different phases of the pandemic. At least 508 million cases and 1.7 million deaths were caused by Omicron-descendent lineages up to 30 June 2023,^15^ driven by convergent evolution towards immune escape at a few specific genetic loci.^16^ However, the large number of deaths during the Omicron wave were due mainly to the high number of cases, and not to increased disease severity associated with Omicron variants.^17^ Specific amino acid substitutions that contributed to the surges in infection and reinfection were acquired through convergent evolution, and included S:R346T, S:K444R/N/M/T, S:V445P, S:N450D, S:L452T, S:N460K, S:F486S and S:F490S.^18^ Here, for example, S:R346T signifies that amino acid R (i.e. arginine) is substituted by T (i.e. threonine) at genetic nucleotide locus 346 of the spike protein (S) coding region. The growth rate advantage of emerging Omicron-descendent lineages over lineages dominant at the time (as identified by a WHO internal growth rate analysis adapted from previous publications)^19^^,^^20^ drove variant replacement, although the date of emergence of new variants and extent of their spread varied between WHO regions (Fig. 3 and Fig. 4). The biological mechanisms that conferred these growth advantages remain unclear, but evidence from computational and laboratory studies suggest they primarily involved successive improvements in immune escape capacity rather than increased intrinsic transmissibility.^21^

## Surveillance for decision-making

The availability of SARS-CoV-2 genome sequence data enables us to trace and, possibly, even forecast the evolutionary trajectory of the virus, thereby providing information on its expected characteristics to guide health-care policy.^22^ The term evolutionary trajectory refers to the genetic changes affecting a virus’s molecular phenotype that potentially confer functional changes that benefit the virus. For instance, an evolutionary trajectory towards higher transmissibility was observed in the early phase of the pandemic when amino acid substitutions in the angiotensin-converting enzyme binding site were associated with an enhanced capacity of SARS-CoV-2 to enter human cells. In addition, in later stages of the pandemic when population immunity was increasing, substitutions accumulating at antibody binding sites conferred immune escape properties. Although the virus was expected to undergo gradual evolution, the sudden and unpredictable genetic shifts that occurred took everyone by surprise. The emergence of variants of concern with large genetic changes was neither predicted nor predictable as they represented a sudden, large evolutionary step. Moreover, variant evolution continues to demonstrate adaptive selection towards higher transmissibility through greater immune evasion as: (i) population immunity increases due to infection and vaccines; and (ii) better access to, and use of, treatments for coronavirus disease 2019 (COVID-19) have reduced morbidity and mortality. In addition, transmission levels remain high globally, partly due to the abandonment of preventive public health and social measures that would have decreased the probability that new variants will emerge either from circulating variants, from the recombination of different viral lineages or from a new genetic constellation.

WHO has adopted a systematic, integrated surveillance approach to monitor the genetic characteristics of viral variants, to analyse patterns of replacement by variants of concern and to assess public health risks. This approach uses the expertise and knowledge of multidisciplinary teams to assess the current state, and predict the evolution, of the SARS-CoV-2 pandemic. A global picture is obtained by linking sources of data and evidence; for example, information on the genetic profile, dynamics and growth rate advantages of different viral variants, and on the breadth and speed of their geographical spread, can be linked to epidemiological data, such as the number of COVID-19 cases, hospitalizations and deaths reported. The analysis is supported by event-based surveillance data from the Epidemic Intelligence from Open Sources initiative, which collates information from official (e.g. health ministries) and unofficial (e.g. news outlets) sources.^23^^–^^25^ Close coordination with WHO regional colleagues, partners (e.g. international experts and public health bodies) and members of the technical advisory group on SARS-CoV-2 virus evolution enables preliminary information to be rapidly shared and assessed, which facilitates the timely detection and investigation of early signals of changes in circulating variants, or in the epidemiological situation in a particular country. The technical advisory group convenes regularly to monitor and assess evidence on the global spread of SARS-CoV-2 variants and on their effect on disease characteristics and clinical countermeasures, such as diagnostics, vaccines, therapeutics, and public health and social measures.^26^

The technical advisory group has established a process for designating SARS-CoV-2 variants as part of an early warning system for emerging variants.^27^ Designated variants of concern and Omicron subvariants that are currently being monitored are listed regularly on WHO’s variant tracking website.^28^ Furthermore, WHO has continued to issue policy briefs that provide guidance on responding to the SARS-CoV-2 epidemic, including one on COVID-19 testing.^29^ The most recent standing recommendations on SARS-CoV-2 were issued on 9 August 2023.^30^ In addition, WHO and the technical advisory group have developed a revised framework for evaluating the public health risk of specific variants that includes the epidemiological indicators essential for sustainable, long-term surveillance, and WHO’s newly developed strategy on global genomic surveillance for pathogens with pandemic and epidemic potential for 2022 to 2032 provides countries with a guide to genomic capacity building.^1^^,^^31^

## Challenges

In the fourth year of the pandemic, it may become challenging to sustain the effective testing and sequencing needed for variant surveillance as countries attempt to balance competing health and socioeconomic priorities. In many countries, routine testing of cases with mild symptoms has already stopped. Moreover, the rapid antigen tests used at home do not contribute to national case reporting and are not amenable to subsequent viral sequencing. Several studies have shown that limited testing and sequencing have consequences for detecting emerging variants. Using data from 189 countries, researchers demonstrated that, in the first two years of the pandemic, 78% of high-income countries sequenced more than 0.5% of COVID-19 cases, whereas only 42% of low- and middle-income countries achieved that level of sequencing.^2^ Furthermore, the study found that, if 0.5% of all cases detected were sequenced within a turnaround time of 21 days, the chance of detecting a new variant in the first 100 cases tested would be only 20%, though this finding largely depends on the testing strategy used. Similar weaknesses in global variant surveillance coverage associated with different testing strategies were also found in other studies.^3^^,^^4^ In one study, mathematical modelling showed that increasing the number of sentinel surveillance sites provides a more accurate way of detecting changes in variant prevalence than simply sequencing more samples nationwide;^4^ in other words, replicating testing at more sites is more efficient than increasing the number of tests per se. Currently, it is recommended that a minimum of 100 samples should be sequenced per 100 000 cases or per day when more than 100 000 cases are sequenced each day.^32^ However, during the first two years of the pandemic, this rate was achieved only by high-income countries and, since January 2023, the rate has declined due to changes in testing strategies.^33^^–^^35^

In light of constrained resources and competing priorities, many countries have largely discontinued SARS-CoV-2 surveillance or have limited surveillance to hospitals and sentinel sites. With the decline in genomic surveillance, there is a risk that a new variant that causes severe disease may be recognized only when hospitals are filled with critical cases and deaths rise. Internationally, many countries made an unprecedented effort to build and enhance genetic and epidemiological surveillance systems – these investments could be maintained and adapted for wider use, thereby ensuring sustainability. In particular, investment in genetic and genomic surveillance could support national pathogen surveillance and elimination programmes, and could help countries prepare for future epidemics and pandemics. The powerful role that can be played by genomic surveillance was exemplified by its widespread use in responding to the SARS-CoV-2 pandemic. Moreover, as the cost of genomic sequencing has fallen, it has become an attainable technology for many more countries. Current and future responses to disease depend on investing in the maintenance of existing surveillance systems and on integrating genomic surveillance for additional pathogens. The aim of the International Pathogen Surveillance Network at WHO’s Berlin-based Hub for Epidemic and Pandemic Intelligence is to help countries maintain the pathogen genomic surveillance capacity built during the SARS-CoV-2 pandemic. In addition, its vision is to ensure that all countries have equitable access to genomics techniques, while bearing in mind that core capacities for disease surveillance and responses must exist in individual countries. This vision can be achieved either by investing in national capacity, or through support and services provided by multilateral or regional networks.

## Future surveillance

The rapid spread of variants of concern may make it difficult for many countries to maintain a high sequencing capacity or to share information over the long term. Only a few countries have the ability to sequence at a level that enables new variants to be detected within 21 days. Also, the cost of sequencing is considerable due to the logistical, material and human resources required. Instead, we propose that future surveillance efforts should focus on four areas: (i) variant surveillance; (ii) variant evolution and phenotype; (iii) variant-induced disease severity; and (iv) integrated pathogen surveillance.

### Variant surveillance

Timely characterization of the circulation and growth rate advantage of current and emerging SARS-CoV-2 variants by countries with sufficient sequencing capacity and linkage to epidemiological data is vital for rapid risk assessment. To facilitate this process, WHO has convened a working group of experts from 25 countries across the six WHO regions that will provide the technical advisory group on SARS-CoV-2 virus evolution with near-real-time global surveillance data to inform risk assessments. 

The routine use of wastewater sequencing has shown promise in several studies from Europe and the United States of America as a sustainable method for estimating the incidence of COVID-19 cases and even of hospitalizations.^36^^–^^38^ A recent publication reported that wastewater-based epidemiological studies in over 150 counties in the United States were able to predict weekly hospital admissions due to COVID-19 one to four weeks in advance.^39^ Although the effectiveness of pathogen identification in wastewater is not new (used for detecting polio and measles), its usefulness for providing an early indication of disease transmission in the community, and even for predicting the potential number of cases and hospitalizations, was highlighted during the SARS-CoV-2 pandemic. With genetic surveillance of wastewater, it is possible to detect the presence of a pathogen, determine the load of a specific genetic variant and monitor trends over time, even when little community-based surveillance is being performed. Consequently, wastewater sequencing can provide an early warning signal and an epidemiological indicator of emerging disease to assist public health decision-making. Although wastewater sequencing cannot replace the contact tracing and community-based surveillance required for individual medical care, it can contribute to population surveillance when little community testing is being performed.

In late 2023, WHO will issue an update to its guidance on environmental surveillance for SARS-CoV-2, highlighting the multiple uses of wastewater surveillance generally and in low-resource settings in particular.

### Variant evolution and phenotype

Coordination among research groups and laboratories is important for providing early, regular insights into the changing genetic and phenotypic characteristics of SARS-CoV-2 variants, such as laboratory findings on their evolutionary trajectories and on phenotypic indicators such as their susceptibility to neutralizing antibodies and COVID-19 severity. Virus isolates can be shared through WHO’s BioHub System, which facilitates the safe and rapid sharing of biological material for cell- and animal-based studies.^40^

### Variant–induced disease severity

Establishing links between SARS-CoV-2 variants circulating in the community and patients’ clinical outcomes is essential for achieving an early understanding of a novel variant’s phenotype and its clinical implications. Countries with a high sequencing capacity and good linkage to patients’ clinical data could play a pivotal role in providing rapid public health risk assessments.

### Integrated pathogen surveillance

Genetic and genomic techniques should become integral elements of national pathogen surveillance. For this to happen, these techniques must be regarded as essential, cost-effective, and sustainable aids to rapid decision-making that will produce a high return on investment by ensuring that public health initiatives are well timed and better targeted. Sustainability may be achieved by expanding the use case of genomics from SARS-CoV-2 to additional pathogens – both pathogens with pandemic potential and local, nationally endemic pathogens. The expansion from pathogen-specific approaches to multi-pathogen, or even so-called pathogen-agnostic approaches is currently underway in several countries. In addition, the expansion of surveillance from individual cases to community sampling, involving for example environmental samples of wastewater or air, are being pioneered as a key component of integrated surveillance. One of the latest technologies with many potential applications in public health surveillance is artificial intelligence. Now that countries are no longer required to report COVID-19 cases, artificial intelligence could be useful for tasks such as identifying and assembling data from health ministry reports, social media and other sources, and for automatically generating epidemiological insights.

## Conclusion

We have reported the main insights gained from the large-scale, global availability of genomic data on SARS-CoV-2 variants early during the pandemic, and have highlighted the continued rapid evolution of the virus as it adapts to the human host and to a changing environment, including increased immunity. In particular, we have reported how linking the genetic features of a variant to its epidemiological and clinical impact was important for the phenotypic and clinical characterization of the variant, and for public health risk assessment. The association between genotype and phenotype remains crucial, as only a few additional amino acid substitutions can profoundly alter the impact of an emerging variant. In addition, we showed how rapidly variants of concern have spread through countries, whole regions and even globally despite many differences in public health measures. Consequently, high sequencing capacity and the sharing of genomic information between countries, networks and regions are essential for pandemic preparedness and for guiding public health responses. We have also demonstrated that the early sharing of genetic and phenotypic data is important, and have suggested ways in which continued variant surveillance can be made more efficient and sustainable. Without continued surveillance, we will not be able to foresee or assess the public health impact of new emerging SARS-CoV-2 variants.
